# Functional and biochemical inflammatory responses to low-dose intra-articular recombinant equine IL-1β: a pilot study

**DOI:** 10.3389/fvets.2025.1746738

**Published:** 2026-01-16

**Authors:** Lindsay Korac, Lindsay St. George, Jennifer MacNicol, Persephone McCrae, L. Jung, N. Golestani, Niel Karrow, Angela Cánovas, Wendy Pearson

**Affiliations:** 1 Animal Biosciences, University of Guelph, Guelph, ON, Canada; 2 School of Health, Social Work and Sport, University of Lancashire, Preston, United Kingdom

**Keywords:** equine joint inflammation, objective gait analysis, prostaglandin E2 (PGE2), subclinical synovitis, synovial fluid biomarkers

## Abstract

Low-dose intra-articular injection of recombinant equine interleukin-1β (reIL-1β) may offer a useful model for studying early onset or subclinical joint inflammation in horses. This pilot study aimed to determine the lowest intra-articular dose of reIL-1β required to produce biochemical evidence of synovitis, and to correlate synovitis biomarkers with functional, upper-body asymmetry parameters. Saline (control) and 50, and 75 ng reIL-1β were injected into the left or right intercarpal joint of three (*n* = 3) horses in a three-way crossover design. Synovial fluid was collected by aseptic arthrocentesis immediately prior to reIL-1β injection (0 h), and at 6-, 12- and 24-h after injection. Synovial fluid was analyzed for inflammatory [prostaglandin E_2_ (PGE_2_) and nitric oxide (NO) and cartilage turnover (glycosaminoglycan (GAG)] biomarkers. Prior to each arthrocentesis, subjective (AAEP score) and objective [inertial measurement unit (IMU)] gait analysis was performed. Asymmetry parameters (MinDiff and MaxDiff) were calculated using IMU data from the poll and pelvis. Mixed model analysis and Spearman correlation coefficient compared biomarker and gait biomechanics data between doses. PGE_2_ concentrations increased significantly (*p* < 0.05) at 6- and 12-h following 50 ng reIL-1β, and at all time points following 75 ng injection, without significantly affecting NO and GAG concentrations (*p* > 0.05). Injection of 75 ng reIL-1β significantly increased poll MinDiff at 6- and 12-h, which was positively correlated with PGE_2_ concentration (ρ = 0.35, *p* < 0.05). Findings support the utility of this lower-dose reIL-1β model for inducing a mild, transient inflammatory response, without overt functional changes, which upholds principles of ethical animal research.

## 1 Introduction

Investigating the inflammatory pathways of the equine joint offers valuable insights into the pathogenesis of diseases such as osteoarthritis (OA), a condition that contributes to substantial economic losses and impaired performance in the equine industry (1). Osteoarthritis in horses is a complex disorder characterized by persistent joint inflammation and progressive cartilage degeneration (2). Experimental models that simulate transient, subclinical synovitis have been described and employed to generate new knowledge about early events contributing to this degenerative process (3–6). These experimental models generally involve intra-articular injection of pro-inflammatory stimuli that produces clinical lameness, but studies have not determined the lowest dose necessary to induce synovitis, and the subsequent effect on functional gait parameters.

Intra-articular injection of lipopolysaccharide (LPS) (4, 5, 7), 1% carrageenan (0.3 mL) (8), or 25 mg of amphotericin B (9) induce acute joint inflammation, usually with pronounced clinical signs. While these models are well-characterized, LPS activates a broad TLR4-mediated response, including both MyD88-dependent and TRIF-dependent pathways, leading to additional interferon-related signaling (10). By contrast, intra-articular recombinant equine interleukin-1β (reIL-1β) at doses ranging from 100 to 200 ng produces self-limiting, more targeted inflammatory response via the MyD88-dependent pathway for a transient inflammation with varying degrees of clinical lameness (4, 11–13). Clinical lameness and increased synovial fluid (SF) biomarker concentrations of inflammation result from intra-articular injection of 100 ng reIL-1β dose, but are not observed when doses of 50 ng are used (4). This distinction formed our rationale for selecting reIL-1β to provide a transient, cytokine-driven model of joint inflammation. Synovial fluid biomarkers, such as nitric oxide (NO) (14, 15), glycosaminoglycan (GAG) (16), and prostaglandin E_2_ (PGE_2_) (17–20), are commonly used to assess cartilage metabolism and joint health. These biomarkers are integral for evaluating the effectiveness of experimental transient inflammatory models, as they provide measurable indicators of inflammatory activity and tissue turnover. Articular inflammation increases NO levels due to inducible nitric oxide synthase (iNOS) activation in response to inflammatory stimuli (21). Elevated NO has been detected in the SF of osteoarthritic joints (22) and the synovial membrane of equine joints with moderate OA (23). GAGs are hydrophilic components of the extracellular matrix, and are released into SF during cartilage turnover (16, 24, 25). The function of GAG in cartilage matrix is to bind water molecules, allowing cartilage to resist compressive forces (26). PGE_2_ is also elevated in joint disease, and it plays a key role in OA by promoting inflammation (27, 28) and stimulating cartilage degradation through provoking an increase in secretion and activation of matrix metalloproteinases (28). It also contributes to pain by sensitizing nerve endings in the joint and amplifying pain signals in the central nervous system (29, 30).

*In vivo* models of OA, which transiently upregulate local mediators of articular inflammation, can show local inflammation and cartilage homeostasis. Typically, they must also produce clinical lameness to facilitate conclusions on functional outcomes, which introduces ethical, and/or economic challenges for conducting equine research. Thus, models that elicit sub/non-clinical changes in functional outcome measures, coupled with relevant biomarker upregulation can be a powerful, minimally invasive experimental strategy to study development, prevention and/or treatment of early-stage OA in horses. Inertial measurement units (IMU) offer a non-invasive, complementary approach to studying changes in intra-articular biomarker concentrations by objectively quantifying changes in upper-body movement asymmetry that may arise following the induction of a transient inflammatory challenge (5). Previous studies have used IMU-based objective gait analysis and physiological SF-derived outcome measures (31–33), during induced inflammatory conditions. However, to the authors' knowledge, objective gait analysis has not been used to evaluate the functional effect(s) of transient, non-clinical synovitis models using reIL-1β through the direct correlation of upper-body asymmetry parameters with SF biomarkers of inflammation. This gap in the literature highlights the opportunity to titrate intra-articular reIL-1β to the lowest concentration required to induce increases in local inflammatory biomarker concentrations, and then to associate biomarker responses with movement asymmetry parameters. Thus, the purpose of the current pilot study was to identify a dose of intra-articular reIL-1β which increases SF PGE_2_, GAG and/or NO, and to associate these increases with functional measures of movement asymmetry. We hypothesized that intra-articular injection of reIL-1β would induce local inflammatory processes within the joint, resulting in elevated concentrations of inflammatory biomarkers in SF, but that the magnitude of this local inflammation would be insufficient to produce detectable changes in upper-body movement asymmetry when compared to baseline.

## 2 Materials and methods

### 2.1 Animals

All experimental procedures and protocols were reviewed and approved by the University of Guelph Animal Care Committee (AUP #4764). Horses were cared for in accordance with the guidelines of the Canadian Council on Animal Care (2017). They were provided with water and grass-hay *ad libitum* in a group-housed turnout yard, with limited access to grass. Three (*n* =3) standardbred mares (10.3 ± 3.2 years; 476.17 ± 28.01 kg) participated in this pilot study. All horses were scored as ≤ 1/5 using the American Association of Equine Practitioners (AAEP) scale, as determined by an experienced assessor (WP).

### 2.2 Intra-articular challenge

Horses were randomly assigned to a 3-way crossover design and received a single, intra-articular injection of 0 ng (control; sterile phosphate-buffered saline), 50 ng, or 75 ng of reIL-1β (Kingfisher Biotech, Inc.) in 500 μL of sterile phosphate-buffered saline, into the left or right intercarpal joint (Supplementary Table S1). The reIL-1β cytokine is species-specific (*Equus caballus*) and was obtained from Kingfisher Biotech, Inc. (Catalog #RP0757E-005; 10 μg/vial). The cytokine was produced in a *Saccharomyces cerevisiae* (yeast) expression system and supplied as purified, lyophilized, biologically active protein suitable for *in vivo* research. Protein was reconstituted in sterile phosphate-buffered saline immediately prior to use, following the manufacturer's instructions. Lot-specific activity was confirmed per the Certificate of Analysis provided by the vendor. Following 48-h wash-out periods, horses were injected in the contralateral intercarpal joint with one of the remaining reIL-1β doses, which was randomly assigned (Supplementary Table S1). The 48-h was selected based on previous studies demonstrating that even higher doses of reIL-1β (100 ng) induce transient synovial inflammation that resolves within approximately 24–48 h (34); thus, a 48-h washout ensured that all clinical and biochemical measures returned to baseline prior to the next injection. Data were collected following the injection of each reIL-1β dose, as described in Section 2.3.

### 2.3 Data collection protocol

#### 2.3.1 Synovial fluid sampling

Hair over the intercarpal joint was clipped, and the skin was desensitized with topical lidocaine/prilocaine cream (Emla cream; Astra Pharmaceutical). After a 15-min contact period, the site was aseptically prepared using 70% isopropyl alcohol and 4% chlorhexidine. A 22-gauge, 1″ sterile needle was inserted into the intercarpal joint, and approximately 1.5 mL of SF was aspirated using a sterile 3 mL syringe. The sample was immediately transferred to a 5 mL heparinized vacutainer. Without removing the needle, a second sterile syringe containing either 0, 50 or 75 μg of reIL-1β in 500 μL sterile saline was attached, and the solution was slowly injected before the needle was withdrawn. Subsequent SF samples were collected aseptically into heparinized vacutainers at baseline (0 h, immediately prior to reIL-1β injection), 6-, 12-, and 24-h post-injection. Samples were placed on ice until collection from all 3 horses was complete, then centrifuged at 900 g for 10 min at room temperature (22 °C). The supernatant was transferred to 1.5 mL tubes containing 10 μL indomethacin (1 mg/mL) to inhibit further prostaglandin synthesis and stored at −80 °C for subsequent analysis of biomarkers associated with joint metabolism (35).

#### 2.3.2 Gait biomechanics

Subjective and objective gait analysis were conducted at each time point (baseline, 6-, 12- and 24-h after reIL-1β injections), immediately prior to SF sampling. Subjective gait assessment was conducted by an experienced researcher (WP) and scored (0–5) using the AAEP scale. Following subjective gait assessment, objective gait analysis was performed using IMU sensors (Delsys Inc., USA), which were fixed to the poll and between the tuber sacrale. Hair at the pelvis site was clipped for consistent placement across time points, with sensors secured using Delsys Adhesive Surface Interface strips (Delsys Inc., USA) and carpet tape. The poll sensor was attached to the halter crown piece. IMU data (370.4 Hz) were collected using EMGWorks Software (Delsys Inc., USA. Version 4.8.0) during four, in-hand trot trials across a 140 ft tarmac runway. Trot speed was measured using a smartphone GPS system secured to a Polar Equine H10 heart rate monitor strap (Polar Equine, Finland). The GPS device recorded instantaneous velocity in kilometers per hour (km/h) at one-second intervals using the Polar Flow App (Polar Equine, Finland) which calculates velocity based on positional data.

### 2.4 Sample and data analysis

#### 2.4.1 Synovial fluid biomarker analysis

PGE_2_ concentration in SF was assessed using a multi-species ELISA kit (Arbor Assays, Mississauga, ON; Catalog #K051-H5). After thawing SF samples to room temperature, aliquots were analyzed in duplicate on antibody-coated 96-well plates in accordance with the kit protocol. A fourth-order polynomial calibration curve (*R*^2^ > 0.99) was established for each plate, and the resulting regression equations were applied to calculate PGE_2_ concentrations.

Nitrite (${\text{NO}}_{2}^{-}$) concentration was quantified using the Griess reaction (36). Undiluted SF was loaded in triplicate into 96-well plates, followed by addition of sulfanilamide (0.01 g/mL) and N-(1)-naphthylethylenediamine hydrochloride (1 mg/mL) prepared in 0.85 g/L phosphoric acid. Plates were read at 530 nm within 5 min of reagent addition. Concentrations were derived from a sodium nitrite standard curve. A best-fit linear regression model (*R*^2^ ≥ 0.99) was generated for each plate and used to calculate nitrite levels.

GAG concentration was determined using a 1,9-dimethylmethylene blue (DMMB) colorimetric assay (36). Synovial fluid samples were thawed to room temperature, diluted at a ratio of 2:13 in dilution buffer (410 mg sodium acetate and 50 μL Tween-20 in 100 mL double-distilled water), and plated in triplicate on 96-well plates. Each well received guanidine hydrochloride (275 mg/mL) and 200 μL of DMMB reagent before absorbance was measured at 530 nm. Concentrations were interpolated from a bovine chondroitin sulfate calibration curve. For each assay plate, a linear regression curve (*R*^2^ > 0.98) was constructed and applied to determine GAG concentrations.

#### 2.4.2 Gait biomechanics

Vertical acceleration data from the poll and pelvis were converted to vertical displacement through double integration and removing the mean from the signal following each integration step (37). Vertical displacement signals were high-pass filtered (Butterworth 4^th^ order) with a cut-off frequency that was adjusted to the stride frequency of each horse (38) and then low-pass filtered (Butterworth 4^th^ order, 30 Hz cut-off) (39). Poll and pelvis vertical displacement signals were stride segmented using the method described by Roepstorff et al. (40) and asymmetry parameters (MinDiff, MaxDiff) were extracted from each stride in accordance with Rhodin et al. (38). Outliers were detected in accordance with Persson-Sjodin (41). This method of deriving poll and pelvis vertical displacement signals, and associated asymmetry parameters, using Delsys Trigno (Delsys Inc., USA) IMU sensors was validated against an optical motion capture system (Qualisys AB, Sweden) in a separate study of 10 horses (St. George, L., unpublished data). The level of agreement between systems was comparable to those reported for previously validated equine gait analysis systems (39, 42) (Supplementary Table S2). Average trot speed per trial was calculated by averaging GPS-derived stride velocity data (km/h) across the defined start and end time for each trial and converting to m/s.

### 2.5 Statistical analysis

Statistical analyses were conducted using RStudio (RStudio, USA, version 2024.09.0). To increase statistical power, asymmetry parameters (MinDiff and MaxDiff) from the poll and pelvis were multiplied by −1, resulting in absolute values and asymmetry independent of sidedness. Linear mixed models (LMMs) were used to examine the effects of timepoint (h) and reIL-1β dose on the measured parameters: trot speed, poll and pelvis asymmetry parameters (MinDiff and MaxDiff), and biochemical outcomes (PGE_2_, NO, and GAG concentrations), with horse included as a random effect to account for repeated measures within individuals. To account for inter-horse variation and in accordance with other studies (43–46), the difference in absolute asymmetry (mm) represented the outcome variable for LMM analysis and was calculated within-horse and condition [control (0 ng), 50 ng and 75 ng reIL-1β] as the difference between the baseline (0 h) value and the associated values at 6-, 12-, and 24-h. Models were fit using the lmer function from the lme4 package, and *p*-values for fixed effects were obtained using Satterthwaite's approximation (47). Estimated marginal means (EMMs) for the interaction between time and dose were calculated using the emmeans package, and pairwise contrasts were performed to compare each timepoint with baseline (0 h) within each reIL-1β dose. Spearman's rank correlation coefficients (ρ) were computed to evaluate potential correlation between biochemical and functional (asymmetry parameters) outcome measures, where significance was identified between timepoint and reIL-1β dose using LMM (48). Correlations were conducted separately for each treatment dose. Statistical significance was defined as *p* < 0.05.

## 3 Results

### 3.1 Synovial fluid biomarkers

The control dose (sterile phosphate-buffered saline) did not significantly alter PGE_2_, GAG, or NO concentrations at any time point (Table 1). Within-treatment changes in GAG and NO from their respective baselines were not significant (*p* > 0.05; Table 1). Conversely, injection of 50 ng of reIL-1β significantly increased PGE_2_ concentrations at 6 h (*p* = 0.01) and 12 h (*p* < 0.05; *p* = 0.03), but not at 24 h (*p* > 0.05) when compared to baseline concentrations (0 h; Table 1). Injection of 75 ng of reIL-1β resulted in significant increases in PGE_2_ concentrations at 6-, 12- and 24 h compared with baseline (0 h (*p* < 0.01; Table 1).

**Table 1:** Estimated marginal (EM) mean (± SD) concentrations of prostaglandin E_2_ (PGE_2_; pg/mL), nitric oxide (NO; μM), and glycosaminoglycans (GAG; μg/mL) in synovial fluid collected from the intercarpal joints of three horses at 0-, 6-, 12-, and 24-h intra-articular injection of 0 ng (control), 50 ng, or 75 ng recombinant equine IL-1β (reIL-1β).

**Dose**	**Timepoint**	**PGE**_**2**_		**NO**		**GAG**	
		**EM Mean** ±**SD**	***p*** **value**	**EM Mean** ±**SD**	***p*** **value**	**EM Mean** ±**SD**	***p*** **value**
0 ng	0	113.92 ± 9.26	/	8.03 ± 2.67	/	41.96 ± 28.39	/
	6	152.32 ± 7.95	0.93	6.93 ± 1.43	0.99	60.25 ± 13.86	0.99
	12	141.85 ± 3.96	0.99	7.19 ± 2.73	0.99	53.07 ± 81.8	0.99
	24	119.63 ± 10.32	0.99	17.57 ± 4.83	0.99	42.75 ± 19.29	0.99
50 ng	0	113.29 ± 17.75	/	5.71 ± 0.26	/	47.32 ± 9.49	/
	6	241.14 ± 3.09	0.01	7.21 ± 1.32	0.99	36.08 ± 41.19	0.99
	12	179.81 ± 5.92	0.03	8.4 ± 1.24	0.99	43.78 ± 37.84	0.99
	24	166.22 ± 11.15	0.22	8.92 ± 0.67	0.99	50.29 ± 11.23	0.99
75 ng	0	140.95 ± 12.67	/	7.56 ± 3.93	/	49.56 ± 40.11	/
	6	359.82 ± 23.77	0.01	10.04 ± 4.64	0.99	49.41 ± 1.66	0.99
	12	324.46 ± 17.76	0.01	13.05 ± 8.81	0.99	75.96 ± 6.89	0.99
	24	294.59 ± 19.39	0.01	9.08 ± 4.46	0.99	56.98 ± 14.97	0.99

*p*-values indicate differences between baseline (0 h) and 6-, 12- and 24-h within each treatment condition [control (0 ng) 50 ng, and 75 ng] as determined by linear mixed model analysis. Significant differences are indicated at *p* < 0.05.

### 3.2 Gait biomechanics

Significant differences in trot speed were not observed across conditions (dose/time point), except at 6 h following 50 ng reIL-1β injection where trot speed significantly increased in comparison to baseline (*p* < 0.05; Table 2). Within-horse AAEP scores did not differ across conditions and were ≤ 1/5. The control dose did not significantly alter poll or pelvis asymmetry parameters (MinDiff or MaxDiff) at any time point relative to baseline (*p* > 0.05; Table 2). Following administration of 75 ng reIL-1β, poll MinDiff increased significantly at 6- and 12-h (*p* < 0.05) compared to baseline, while changes in Poll MaxDiff were non-significant across all time points (*p* > 0.05; Table 2). Changes in pelvis asymmetry parameters also were non-significant across all timepoints following 75 ng reIL-1β dose (*p* > 0.05), except at 12 h, where significant decreases in MinDiff (*p* = 0.04) and MaxDiff (*p* = 0.01) were observed (Table 2). Following injection of 50 ng reIL-1β, MaxDiff significantly decreased at 6 h for the poll (*p* < 0.05), and at 12- and 24 h the pelvis (*p* < 0.01) when compared to baseline, but MinDiff did not significantly differ from baseline values across timepoints (*p* > 0.05; Table 2).

**Table 2:** Estimated marginal (EM) mean difference (± SD) between corresponding baseline (0 h) and treatment [0 ng (control), 50 ng, or 75 ng recombinant equine IL-1β (reIL-1β)] conditions at 6-, 12-, and 24-h and associated *p*-values for poll and pelvis asymmetry parameters (MinDiff and MaxDiff; mm) and trot speed (m/s) from three horses.

**Dose**	**Timepoint**	**Poll MinDiff**		**Poll MaxDiff**		**Pelvis MinDiff**		**Pelvis MaxDiff**		**Trot speed**	
		**EM Mean** ±**SD**	***p*** **value**	**EM Mean** ±**SD**	***p*** **value**	**EM Mean** ±**SD**	***p*** **value**	**EM Mean** ±**SD**	***p*** **value**	**EM Mean** ±**SD**	***p*** **value**
0 ng	6 h	6.72 ± 4.01	0.23	−2.08 ± 3.49	0.85	−0.61 ± 1.52	0.93	−2.34 ± 1.43	0.25	−0.20 ± 0.17	0.47
	12 h	−1.84 ± 3.44	0.88	−3.54 ± 3.00	0.49	4.02 ± 1.41	0.13	3.27 ± 1.34	0.40	0.06 ± 0.14	0.93
	24 h	−0.75 ± 3.69	0.98	−5.63 ± 3.21	0.19	−0.38 ± 1.43	0.97	0.10 ± 1.35	0.99	−0.14 ± 0.15	0.67
50 ng	6 h	1.70 ± 3.28	0.88	−7.66 ± 2.85	0.02	−0.49 ± 1.42	0.95	−3.03 ± 1.34	0.07	0.48 ± 0.13	0.01
	12 h	4.71 ± 3.25	0.34	−6.44 ± 2.82	0.06	0.19 ± 1.39	0.99	−5.09 ± 1.31	< 0.01	−0.11 ± 0.13	0.75
	24 h	2.10 ± 3.45	0.84	−4.25 ± 2.99	0.35	0.75 ± 1.37	0.87	−5.39 ± 1.30	< 0.01	0.12 ± 0.14	0.70
75 ng	6 h	12.56 ± 3.64	< 0.01	1.42 ± 3.16	0.92	−2.09 ± 1.38	0.30	−2.66 ± 1.30	0.11	−0.22 ± 0.15	0.31
	12 h	9.89 ± 3.65	0.02	2.13 ± 3.22	0.82	−3.46 ± 1.40	0.04	−3.86 ± 1.32	0.01	0.09 ± 0.15	0.83
	24 h	1.07 ± 3.76	0.96	3.11 ± 3.27	0.64	0.83 ± 1.38	0.85	−2.14 ± 1.30	0.24	−0.48 ± 0.15	0.10

Significant differences are indicated at *p* < 0.05.

### 3.3 Correlation between biomarkers and gait biomechanics

Correlations between PGE_2_ and movement asymmetry parameters (poll and pelvis MinDiff and MaxDiff) were evaluated using Spearman rank correlation coefficient, given the significant effect (*p* < 0.05) of reIL-1β dose and timepoint that was determined using LMM. For the 75 ng reIL-1β dose, a significant positive correlation between PGE_2_ concentration and poll MinDiff (ρ = 0.352, *p* < 0.01; Table 3) was observed. Conversely, for 50 ng and 75 ng reIL-1β doses, significant negative correlations were observed between PGE_2_ concentration and pelvis MaxDiff (50 ng: ρ = −0.273, *p* < 0.01, 75 ng: ρ = −0.421, *p* < 0.01) and pelvis MinDiff (50 ng: ρ = −0.215, *p* < 0.01, 75 ng: ρ = −0.311, *p* < 0.01; Table 3). Significant correlations between PGE_2_ and asymmetry parameters were not observed for the control condition (Table 3).

**Table 3:** Spearman correlation coefficients (ρ) between prostaglandin E_2_ (PGE_2_; pg/mL) concentrations and absolute poll and pelvis asymmetry parameters (MaxDiff and MinDiff; mm) from three horses following intra-articular injection of 0 ng (control), 50 ng, or 75 ng recombinant equine IL-1β (reIL-1β) into the intercarpal joint.

**Region**	**Parameter**	**Dose**	**Spearman ρ**	***p* value**
Poll	MaxDiff	0	−0.15	0.14
		50	−0.04	0.71
		75	0.05	0.62
	MinDiff	0	0.02	0.88
		50	0.15	0.11
		75	0.35	< 0.01
Pelvis	MaxDiff	0	−0.03	0.67
		50	−0.27	< 0.01
		75	−0.42	< 0.01
	MinDiff	0	−0.09	0.23
		50	−0.22	< 0.01
		75	−0.31	< 0.01

## 4 Discussion

This pilot study aimed to evaluate the effect of 50 ng and 75 ng doses of intra-articular reIL-1β on selected SF biomarkers (PGE_2_, GAG and/or NO), and objective movement asymmetry parameters over a 24-h period. Our findings demonstrate significant increases in equine SF PGE_2_ concentrations at 6- and 12-h post intercarpal injection of 50 ng and 75 ng reIL-1β, which persists for up to 24 h following 75 ng injection. Conversely, NO and GAG concentrations were not significantly altered by the reIL-1β doses tested in the present study. In addition, significant increases in poll MinDiff were observed at 6- and 12-h following intercarpal injection of 75 ng reIL-1β ng when compared to baseline measurements, a change that was positively correlated with PGE_2_ concentration. Taken together, these findings partially support the hypothesis, as significant differences in PGE_2_ concentrations and movement asymmetry parameters were observed following intra-articular injection of 50 ng and 75 ng reIL-1β.

Clinical experimental models of equine synovitis typically use doses upwards of 100 ng of reIL-1β to produce articular inflammation (4) and overt lameness (4, 49). However, our approach aimed to produce a subclinical, physiologically detectable inflammatory response using lower doses of reIL-1β. Notably, 50 ng and 75 ng reIL-1β doses elicited significant elevations in PGE_2_ concentrations indicating that an inflammatory response was initiated. PGE_2_ is a key inflammatory mediator that is specifically upregulated in response to reIL-1β stimulation, particularly within articular tissues (50). Elevated SF PGE_2_ concentrations of 263 pg/mL ± 132.3 and 656.8 pg/mL have respectively been reported in horses following an exercise bout (36) and in osteoarthritic horses (51) where it accumulates locally at the site of inflammation (52–54). The PGE_2_ concentrations observed here are lower than those previously reported for osteoarthritic horses and align more with those related to an exercise-induced inflammatory response. These findings highlight the potential of this lower-dose reIL-1β model as a more refined experimental approach for studying joint inflammation in horses (55).

Interestingly, intra-articular injection of 50 and 75 ng reIL-1β did not produce significant changes in equine SF GAG concentrations. Degradation of the extracellular matrix (ECM) of cartilage is a hallmark of OA, and when cartilage breaks down, GAGs are released into the SF (56). However, the difference in SF GAG concentrations between healthy horses and those with joint disease differs across experimental studies (16, 23). In one study, intercarpal injection of 100 ng of reIL-1β significantly increased equine SF GAG concentrations at 24- and 48-h post-injection (4). A potential explanation for the non-significant changes in GAG concentration observed here, is that lower-doses of intra-articular reIL-1β did not cause measurable ECM degradation within the 24-h study period. It is also possible that this pilot study was underpowered to detect subtle GAG responses to a lower dose reIL-1β challenge. Future studies are required to assess the effect of various intra-articular reIL-1β doses on SF GAG concentrations in a larger sample of horses. This will enable researchers to determine the lowest dose at which a significant GAG response is measurable.

Like GAG, our study did not identify a significant effect of 50 ng or 75 ng intra-articular reIL-1β on equine SF NO concentration. ReIL-1β provokes chondrocytes to produce catabolic matrix metalloproteinases that function to accelerate ECM turnover. This process contributes to the activation of iNOS, which facilitates NO formation (57, 58). Previous studies have demonstrated elevated NO levels in the synovial membrane of equine joints affected by OA (23). However, a study evaluating the effect of transient, exercise-induced joint inflammation on SF inflammatory biomarkers in horses reported no significant changes in NO concentration across the studied time-course (36). This finding is consistent with the results of our study and supports the postulation that cartilage degradation did not occur in response to the low-dose reIL-1β injection studied here (36, 59). Our findings indicate that NO and GAG concentrations in equine SF are not elevated following intercarpal injection of 50 ng or 75 ng reIL-1β, and future studies are needed to determine reIL-1β concentrations that can influence NO accumulation in SF.

Despite the non-significant alterations in equine SF, GAG, and NO concentrations that were observed in this small sample of horses, the significant increases in PGE_2_ concentration indicate an inflammatory response to both 50 ng and 75 ng doses of reIL-1β. As such, the proposed model effectively induces transient synovitis without eliciting detectable cartilage degradation, as reflected by stable GAG and NO concentrations. Further studies are required to evaluate additional SF inflammatory biomarkers, which will enable us to gain a more comprehensive understanding of the inflammation resolution response(s) to this model.

In addition to quantifying the effect of intra-articular reIL-1β on inflammatory biomarkers, this study sought to determine whether subsequent functional changes were elicited, using objective upper-body movement asymmetry parameters. The poll MinDiff parameter is a quantitative measure of asymmetry, that indirectly describes differences in vertical loading between the left and right forelimbs (60). Here, significant increases in poll MinDiff were observed following injection of 75 ng reIL-1β at 6- and 12-h. These increases in poll MinDiff were less pronounced at 12 h were no longer significantly different from baseline at 24 h post-injection. This finding, along with the positive correlation between Poll MinDiff and PGE_2_ concentration, aligns with the predicted transient inflammatory response created by intercarpal injection of reIL-1β (36). In addition to acting as a potent inflammatory mediator, PGE_2_ also contributes to peripheral sensitization of nociceptors within synovial and periarticular tissues, lowering the activation threshold of sensory afferents and amplifying pain perception (17, 28, 61). In horses, elevated PGE_2_ in SF has been linked to lameness and experimentally induced joint pain, reflecting its role in generating hyperalgesia at the site of inflammation (29, 54, 62). Thus, the significant rise in PGE_2_ concentrations following 75 ng reIL-1β injection may have enhanced local nociceptive signaling within the treated intercarpal joint, giving rise to subtle discomfort that was sufficient to elicit compensatory offloading of the affected forelimb (29). Importantly, the differences between Poll MinDiff at baseline and at 6- and 12-h timepoints fall within the reported range of inter-session variability for this parameter in healthy horses (63, 64). As such, it is possible that significant increases in head movement asymmetry are the result of biological variation between measured timepoints, rather than a causal effect of intercarpal 75 ng reIL-1β treatment. Still, these findings justify future work to establish cause and effect relationships between biochemical and biomechanical data and to determine whether the observed changes in head movement are clinically meaningful.

Slight, albeit significant, decreases in pelvis MinDiff and MaxDiff were observed at 12 h following 75 ng reIL-1β treatment and these asymmetry parameters were negatively correlated with PGE_2_ concentration. Again, these differences fall within reported ranges of inter-session variability for these pelvic asymmetry parameters in healthy Thoroughbred and sport horses (63, 64). Similarly, the slight, but significant, decreases in poll and pelvis MaxDiff that were observed at various timepoints following the 50 ng reIL-1β treatment were also in accordance with inter-session variability ranges (63, 64). Therefore, the observed changes in pelvic asymmetry suggest that intercarpal injection of 50 and 75ng reIl-1β is not sufficient to elicit compensatory adaptations in hindlimb movement (65). However, future work in a larger sample of horses is required to confirm this interpretation.

## 5 Limitations

There are limitations that must be considered when interpreting the findings of this pilot study. The same investigator administered the intra-articular injections and performed the lameness evaluations and future work should consider incorporating blinding of evaluators to treatment allocation. A small sample size was employed in this cross-over study, and future work with larger sample sizes are needed to confirm our findings. Our investigation also focused on a limited set of time points (6-, 12-, and 24-h), so the effects of prolonged inflammation or recovery beyond 24 h were not assessed here and should be further evaluated. While the biochemical markers examined (PGE_2_, NO, and GAG) are well-established in the literature, additional mediators, such as the pro-resolution molecule resolvin D1, could offer further insight into the mechanisms of joint inflammation, its resolution, and potential influence on gait biomechanics (66, 67). Finally, trot speed was not standardized within- and between-sessions and has a known effect on movement asymmetry parameters (68–70). However, differences in trot speed between conditions/timepoints were generally non-significant and exhibited low standard deviations. Still, future studies may build on this work by controlling for speed.

## 6 Conclusion

This study demonstrates that intercarpal injection of 50 ng and 75 ng of recombinant equine IL-1β is sufficient to significantly increase synovial fluid PGE_2_ concentration, which persists for up to 24 h following 75 ng injection. In addition, significant increases in poll MinDiff at 6- and 12-h were observed following intercarpal injection of 75 ng reIL-1β ng when compared to baseline measurements, a change that was positively correlated with PGE_2_ concentration. Conversely, NO and GAG concentrations were not significantly altered by the reIL-1β doses studied here, which suggests that this acute model does not result in measurable cartilage degeneration. These findings provide preliminary data to support the use of this lower-dose IL-1β model for investigating inflammatory mechanisms under ethically refined conditions, which may be particularly relevant for future pre-clinical research in equine inflammatory conditions.

## Data Availability

The original contributions presented in the study are included in the article/Supplementary material, further inquiries can be directed to the corresponding author.
