# Supplementary material for: Functional and biochemical inflammatory responses to low-dose intra-articular recombinant equine IL-1β: a pilot study
Source: Front Vet Sci. 2026 Jan 16;12:1746738. doi: 10.3389/fvets.2025.1746738 (PMC12855077; doi:10.3389/fvets.2025.1746738)
Supplement: Supplementary file 1 [file Data_Sheet_1.pdf]

Supplementary Material

1 Supplementary Figures and Tables

Supplementary Table 1: Treatment allocation by horse, age, and injection site. Each horse received three intra-articular injections of recombinant equine IL-1β (reIL-1β) at doses of 0, 50, and 75 ng/mL into the intercarpal joint in a randomized crossover design. Superscripts denote the injection site: *a* = left intercarpal joint; *b* = right intercarpal joint.

		Treatment (ng/mL)					
**Horse**	**Age**	**Dose 1**		**Dose 2**		**Dose 3**	
1	14	0	L[a]	50	R[b]	75	L
2	8	50	L	75	R	0	L
3	9	75	L	0	R	50	L

[a] left intercarpal joint; [b] right intercarpal joint

Supplementary Table 2. Summary of measurement agreement statistics from the validation study of n=10 horses for asymmetry parameters (MinDiff, MaxDiff) from the poll and pelvis calculated using IMU and OMC data.

**Variable**		**N (strides)**	**Bias**	**Upper LoA**	**Lower LoA**	**ICC**
Pelvis MaxDiff	Per Stride	106	0.6	9.9	-8.6	-
	Per Trial	10	0.9	5.1	-3.3	0.97
Pelvis MinDiff	Per Stride	106	-0.5	11.2	-12.3	-
	Per Trial	10	-0.6	10.6	-11.8	0.93

Poll MaxDiff	Per Stride	88	0.12	17.38	-17.13	-
	Per Trial	10	1.02	11.67	-9.64	0.97
Poll MinDiff	Per Stride	92	0.07	15.42	-15.28	-
	Per Trial	10	-1.03	8.70	-10.76	0.96
